# The Expression of the Cancer-Associated lncRNA *Snhg15* Is Modulated by EphrinA5-Induced Signaling

**DOI:** 10.3390/ijms22031332

**Published:** 2021-01-29

**Authors:** Daniel Pensold, Julia Gehrmann, Georg Pitschelatow, Asa Walberg, Kai Braunsteffer, Julia Reichard, Amin Ravaei, Jenice Linde, Angelika Lampert, Ivan G. Costa, Geraldine Zimmer-Bensch

**Affiliations:** 1 Division of Functional Epigenetics, Institute of Zoology (Biology 2), RWTH Aachen University, Worringerweg 3, 52074 Aachen, Germany; pensold@bio2.rwth-aachen.de (D.P.); georg.pitschelatow@rwth-aachen.de (G.P.); asa.walberg@rwth-aachen.de (A.W.); kai.braunsteffer@rwth-aachen.de (K.B.); reichard@bio2.rwth-aachen.de (J.R.); jenice.linde@rwth-aachen.de (J.L.); 2 RWTH Aachen Medical Faculty, Institute for Computational Genomics, 52074 Aachen, Germany; julia.gehrmann@rwth-aachen.de (J.G.); ivan.costa@rwth-aachen.de (I.G.C.); 3 Research Training Group 2416 Multi Senses—Multi Scales, RWTH Aachen University, 52074 Aachen, Germany; alampert@ukaachen.de; 4 Department of Neurosciences and Rehabilitation, Section of Medical Biochemistry, Molecular Biology and Genetics, University of Ferrara, 44100 Ferrara, Italy; rvamna@unife.it; 5 RWTH Aachen Medical Faculty, Institute of Physiology, 52074 Aachen, Germany

**Keywords:** proliferation, migration, glioma, lncRNA, triplex target DNA sites, EphA2

## Abstract

The Eph receptor tyrosine kinases and their respective ephrin-ligands are an important family of membrane receptors, being involved in developmental processes such as proliferation, migration, and in the formation of brain cancer such as glioma. Intracellular signaling pathways, which are activated by Eph receptor signaling, are well characterized. In contrast, it is unknown so far whether ephrins modulate the expression of lncRNAs, which would enable the transduction of environmental stimuli into our genome through a great gene regulatory spectrum. Applying a combination of functional in vitro assays, RNA sequencing, and qPCR analysis, we found that the proliferation and migration promoting stimulation of mouse cerebellar granule cells (CB) with ephrinA5 diminishes the expression of the cancer-related lncRNA *Snhg15.* In a human medulloblastoma cell line (DAOY) ephrinA5 stimulation similarly reduced *SNHG15* expression. Computational analysis identified triple-helix-mediated DNA-binding sites of *Snhg15* in promoters of genes found up-regulated upon ephrinA5 stimulation and known to be involved in tumorigenic processes. Our findings propose a crucial role of *Snhg15* downstream of ephrinA5-induced signaling in regulating gene transcription in the nucleus. These findings could be potentially relevant for the regulation of tumorigenic processes in the context of glioma.

## 1. Introduction

Eph receptors represent the largest subfamily of receptor tyrosine kinases (RTKs), initially isolated from an erythropoietin-producing human hepatocellular carcinoma line [1]. By interacting with their cognate ligands, the ephrins (Eph receptor-interacting ligands), Eph receptors mediate important aspects of embryogenesis and the development of numerous tissues, including the brain [1]; such as proliferation, cell adhesion, axon guidance, cell migration, and others [1,2,3,4,5,6,7]. Moreover, Eph/ephrin signaling impacts multiple aspects of cancer development and progression, affecting the proliferation and migration of cancer cells as well as tumor invasiveness, angiogenesis, and metastasis [8]. 

Eph receptor and ephrin function play a crucial role in gliomas, which account for one of the most aggressive and common primary tumors (~30%) of the central nervous system (CNS) [1]. Gliomas include astrocytoma, oligodendroglioma, ependymoma, medulloblastoma, and glioblastoma, which differ in their origins, aggressiveness, and progression. Despite enormous effort in developing novel therapeutic strategies, the different types of gliomas still represent one of the main medical challenges with poor patient’s prognosis [1]. Dysregulation of several members of the Eph/ephrin family, being localized on the surface of tumor cells, tumor vasculature, and glioma stem cells, was linked to patients’ outcome [1]. The ligands and receptors can promote and inhibit tumorigenicity depending on the downstream forward and reverse signaling generated. Hence, to dissect their potential as therapeutic targets, downstream signaling, and effects on gene expression have to be better understood. 

Eph receptor forward signaling triggered by binding of cognate ligands drives the activation of numerous pathways that signal to the nucleus, such as the PI3K-Akt/PKB and the Ras/MAPK pathway [9]. This induces gene expression changes that mediate the physiological responses. What is unknown so far is, whether Eph/ephrin signaling can modulate the expression of long non-coding RNAs (lncRNAs). This is of particular importance, as lncRNAs recently came to the fore as diagnostic markers of glioma, being involved in glioma initiation and progression [10]. Moreover, the mechanistical spectrum of lncRNA function is enormously diverse, ranging from transcriptional regulation, splicing mediation, post-transcriptional processing, functions as sponge and precursor of miRNAs, as well as chromatin remodeling via triple-helix-mediated DNA-RNA interactions [11,12,13,14,15]. Being classified as signal, scaffold, decoy, and enhancer, based on their targeting mechanism [14], lncRNAs regulate gene expression at transcriptional, post-transcriptional, and epigenetic levels [16,17]. 

An important lncRNA, which has been associated with proliferation and migration regulation in different human cancer tissues, is the small nucleolar RNA host gene 15 (*SNHG15*). Dependent on the cancer type, *SNHG15* has been found overexpressed or silenced, functioning either as cancer- and metastasis-promoting lncRNA, or as tumor-suppressor [13]. *SNHG15* has been demonstrated to affect the expression of genes with roles in cell proliferation, migration, and survival [18]. Although *SNHG15* has been described to serve as a competitively endogenous RNA (ceRNA) sponging miRNA in human cancers [19], it was also reported that *SNHG15* is seen at much higher levels in the nucleus than in the cytoplasm [20]. Moreover, it was reported that *Snhg15* functions in the nucleus, e.g., by interacting with EZH2, the core enzyme of the polycomb repressor complex 2 that catalyzes repressive trimethylation of H3K27me3 [20]. Importantly, *Snhg15* is known to be involved in mediating the responsiveness to environmental stimuli [21]. Of note, the expression of numerous lncRNAs was reported to be modulated by environmental stimuli. For this, lncRNAs are proposed to play a key role in environmental sensing, e.g., by conveying between membrane-bound receptors and gene expression regulation [22,23], which could contribute to phenotypic diversity of glioblastoma subclasses.

Here we investigated whether ephrinA5 modulates the expression of lncRNAs in immortalized cerebellar granule (CB) cells, which are often used as a medulloblastoma model based on the observation that medulloblastoma can arise from CB progenitor cells [24,25]. We found that ephrinA5-induced stimulation triggers changes in the expression of lncRNAs, snRNAs, miRNAs, and other non-annotated transcripts. Among the lncRNAs, we identified *Snhg15* as significantly reduced in expression in response to ephrinA5 stimulation in CB cells. An ephrinA5-Fc triggered reduction in *SNHG15* was further observed in DAOY cells, a human medulloblastoma cell line [26]. *Snhg15* is known to be involved in proliferation and migration regulation in various human cancer types [13]. Interestingly, ephrinA5 stimulation results in increased proliferation and migration of CB cells. These physiological responses were found to be accompanied by transcriptional remodeling of associated genes. We determined the putative binding sites of *Snhg15* to promoter regions of numerous genes identified as up-regulated in expression upon ephrinA5 stimulation. This suggests that *Snhg15* could be involved in the transcriptional regulation of these genes by preventing or recruiting the binding of transcription factors, or chromatin remodelers. 

## 2. Results

### 2.1. EphrinA5-Fc Stimulation Induces Changes in the Expression of Non-Coding RNAs in Addition to Protein-Coding Genes

**Figure 1:**
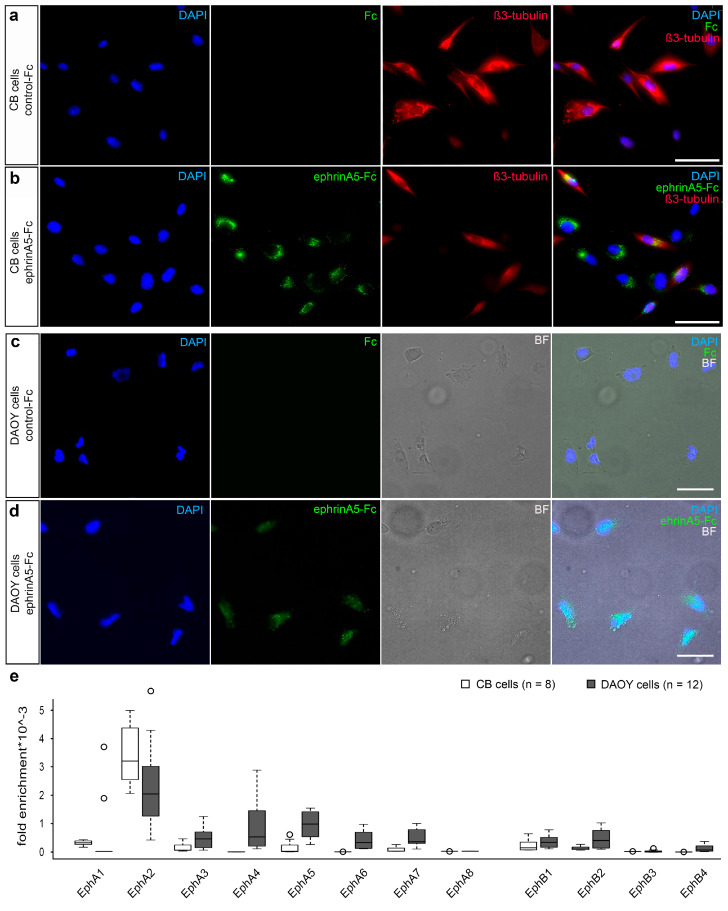
Expression of Eph receptors and illustration of ephrinA5-Fc binding sites in CB and DAOY cells. (**a**,**b**) CB cells were tested for binding of recombinant control-Fc (**a**) and recombinant ephrinA5-Fc (**b**), both clustered with an Alexa488 conjugated anti-human IgG antibody (green, second panel). CB cells were additionally stained using DAPI (blue, first panel) and an antibody directed against ß3-tubulin (red, third panel). The overlay of all channels is depicted in the fourth panel. (**c**,**d**) DAOY cells were treated with control-Fc (**c**) or recombinant ephrinA5-Fc (**d**), both clustered with an Alexa488 conjugated anti-human IgG (green, second panel). In addition to DAPI nucleus staining (blue, first panel), brightfield is depicted (third panel) as well as the overlay of all channels (fourth panel). (**e**) Quantitative real time PCR analysis of expression of *EphA* and *EphB* receptors depicted as fold enrichment relative to the reference genes *Rps29* and *RPS18* (*n* = 8 for CB cells and *n* = 12 for DAOY cells. N = 4 biological replicates). Scale bar: 50 µm.

To test whether ephrinA5 triggers changes in lncRNA expression, we stimulated immortalized CB cells with recombinant ephrinA5-Fc, or Fc as control. Both were clustered with an Alexa488-labelled anti-human IgG antibody to achieve Eph receptor activation according to previous studies, and at the same time allowing for visualization of ephrinA5-Fc-binding sites [2,3]. Indeed, we observed Alexa488-labelled ephrinA5-Fc binding for the majority of the CB cells (Figure 1a,b). EphrinA5 is known to interact with numerous Eph receptors, including EphA receptors in addition to EphB2 [2,27]. To determine which of the receptors is expressed by CB cells and hence mediates ephrinA5 binding and signaling, we performed qPCR analysis. Our experiments revealed that among all ephrinA5-interacting receptors, EphA2 displays the most prominent expression in CB cells (Figure 1e). Very similar results were found when stimulating DAOY cells (ATCC^®^ HTB-186™), representing human cerebellar medulloblastoma cells, with ephrinA5-Fc using the same protocol (Figure 1c,d). Similar to CB cells, ephrinA5-Fc-binding sites were observed for the majority of DAOY cells, and the EphA2 receptor was found most prominently expressed (Figure 1c–e). Of note, ephrinA5 stimulation caused an increased expression of EphA2 in DAOY cells (Supplementary Figure S1a), which is interesting as EphA2 overexpression was observed in numerous cancer cells including human glioblastoma multiform (GBM) tumors [28].

**Figure 2:**
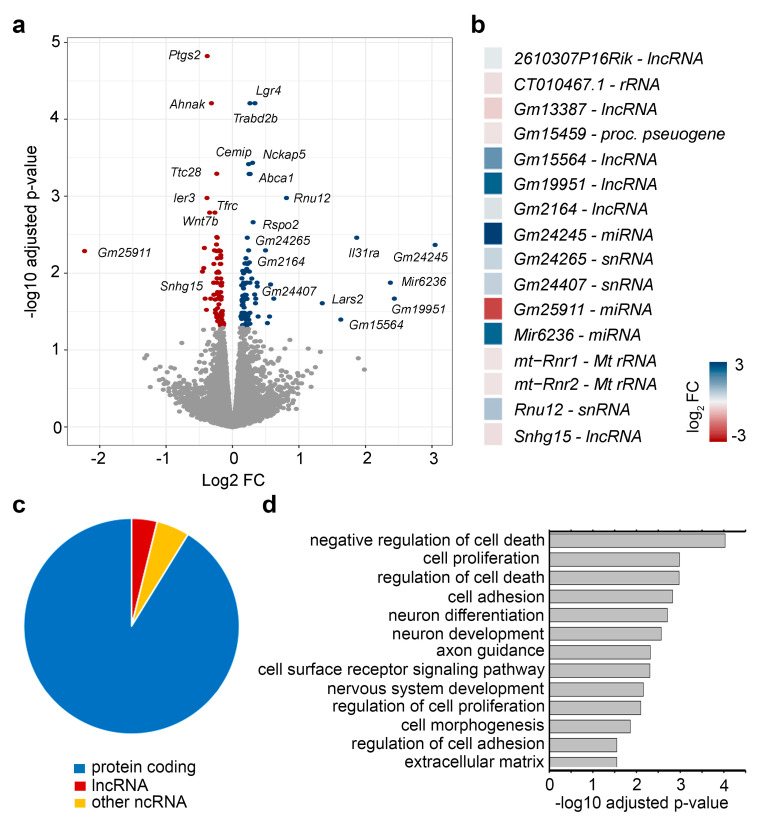
Differential gene expression analysis of ephrinA5-Fc-treated CB cells versus controls (Fc) determined by RNA sequencing of nuclear-enriched and ribosomal-depleted RNA. (**a**) Volcano plot illustrates the genes identified as significantly up- and down-regulated upon ephrinA5-Fc stimulation. The negative log10 of the adjusted *p*-value is plotted against the log2 fold change of each gene. Significantly up-regulated genes are depicted in blue, and significantly down-regulated genes are illustrated in red. Sequencing was performed as N = 3 biological replicates per condition. (**b**) Heatmap of differentially expressed non-coding RNAs. (**c**) Pie chart diagram depicting the proportions of protein-coding versus non-coding RNAs found as differentially expressed. (**d**) Bar plot depicting significant gene ontology terms for the differentially expressed genes after ephrinA5-Fc stimulation.

Next, we aimed to profile the transcriptional changes induced by ephrinA5-triggered signaling by the use of the CB cell model. To this end, CB cells were stimulated with pre-clustered ephrinA5-Fc or Fc protein for 24 h. Afterwards, we isolated nuclear-enriched RNA and performed RNA sequencing to determine the differentially expressed RNAs including mRNAs as well as non-coding RNAs. The PCA plot illustrates the segregation of control-Fc- and ephrinA5-Fc-treated samples (Supplementary Figure S1b). Moreover, we found that in addition to protein-coding genes, a high proportion of the reads were annotated for lncRNA species (Supplementary Figure S1c), supporting the successful enrichment of nuclear RNA species. Although protein-coding genes represented the largest fraction among all detected gene species, different lncRNA species were likewise detected (Supplementary Figure S1d). Differential gene expression analysis revealed that 182 different genes were significantly increased or decreased in their expression in CB cells upon ephrinA5-Fc stimulation (Figure 2a,b, Supplementary Figure S1e), most of which represent protein-coding genes (Figure 2c). However, we also determined ncRNAs to be differentially expressed in response to ephrinA5-Fc stimulation (Figure 2c), being both increased or diminished in their expression (Figure 2b). 

Next, we performed Gene Ontology (GO) analysis with the set of genes found to be differentially expressed upon ephrinA5-Fc stimulation in CB cells. We determined a significant enrichment of proliferation, cell death and cell adhesion, extracellular matrix, cell surface receptor signaling and neuronal development related genes (Figure 2d). This is consistent with the reported functions of ephrinA5 and EphA2, influencing proliferation, cell death, cell adhesion, and migration in diverse cancer types including glioma [29] as well as during nervous system development [4,7,30,31,32,33]. EphrinA5 induced signaling regulates proliferation and differentiation of neuronal progenitors, neuronal migration, and axonal guidance in diverse parts of the developing brain [4,7,30,31,32,33]. Together, this suggests that physiologically relevant transcriptional changes induced by ephrinA5-Fc treatment in CB cells were profiled by analyzing nuclear-enriched RNA. 

### 2.2. EphrinA5-Fc Treatment of CB Cells Stimulates Their Motility and Proliferation

**Figure 3:**
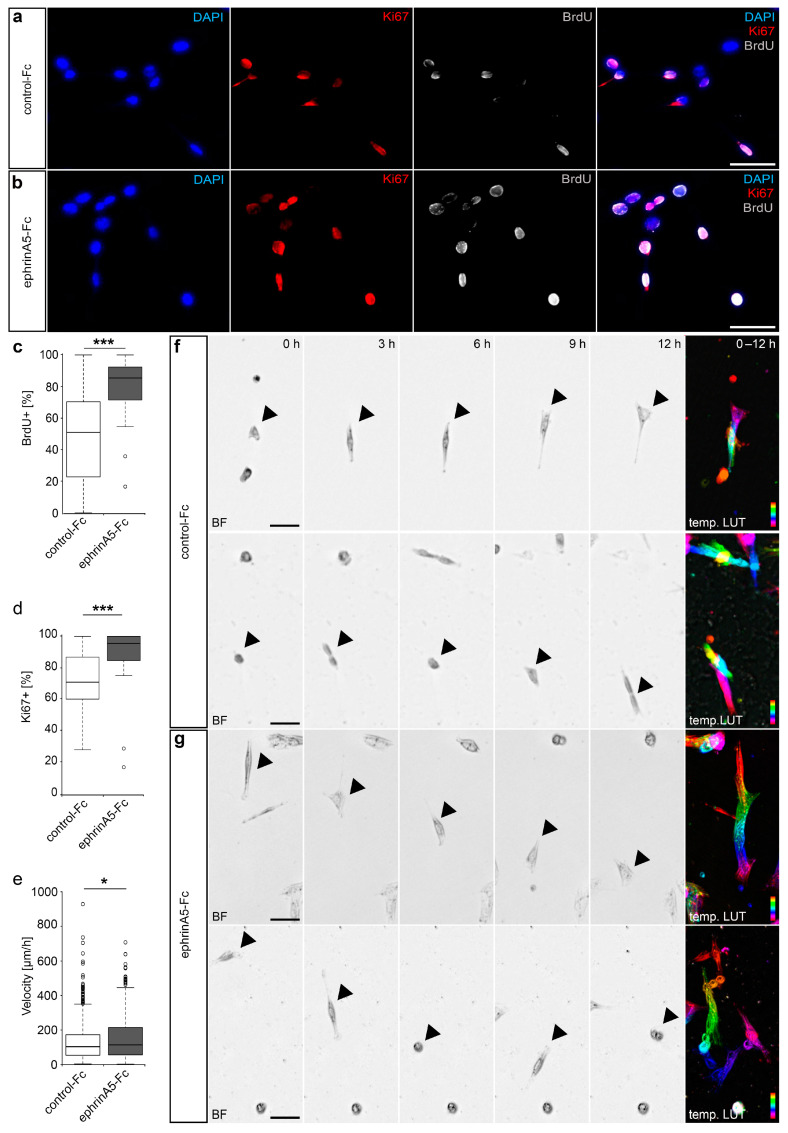
EphrinA5-Fc stimulation results in increased proliferation and motility of CB cells. (**a**,**b**) CB cells treated with control-Fc (**a**) or recombinant ephrinA5-Fc (**b**), both clustered with an Alexa488 conjugated anti-human IgG antibody, were stained with DAPI (blue, first panel) and antibodies directed against Ki67 (red, second panel) and BrdU (grey, third panel). The overlay of all channels is depicted in the fourth panel. (**c**) Quantitative analysis of the proportion of BrdU positive cells, with BrdU pulses given one hour prior to fixation of cells. (**d**) Quantitative analysis of the proportion of Ki67 positive cells (*n* = 645 cells for control-Fc and *n* = 451 cells for ephrinA5-Fc; N = 3 experiments). (**e**–**g**) Live cell imaging analysis of migrating CB cells treated with ephrinA5-Fc and control-Fc revealed increased velocities upon ephrinA5-Fc stimulation as quantified in (**e**); *n* = 618 cells for control-Fc and *n* = 576 cells for ephrinA5-Fc; N = 3 experiments). (**f**,**g**) Representative brightfield images of temporal sequences illustrating the migratory route of selected CB cells after treatment with control-Fc (**f**) and ephrinA5-Fc (**g**), depicting frames at 0 h, 3 h, 6 h, 9 h, and 12 h of the respective cell track. The last panel in (**f**,**g**) represents the color-coded migratory distances within the depicted 12 h time period (temporal LUT), indicating the starting point (red, top) and the end point (magenta, bottom) of the respective cell. Two-tailed Student’s *t*-test, * *p* < 0.05, *** *p* < 0.001. Scale bars 50 µm. BF: brightfield, temp. LUT: temporal LUT, Ctrl: control.

Next, we aimed to verify the observed transcriptional changes at a functional level by investigating the physiological responses of CB cells toward ephrinA5 stimulation. Based on the GO analysis and described functions of Eph/ephrins in the cancer-context, we here focused on analyzing the effects on CB cell proliferation and migratory activity. To determine whether ephrinA5-Fc impacts proliferation, we stimulated cells with pre-clustered ephrinA5-Fc for 5 h and then applied the thymidine analogue Bromodeoxyuridine (BrdU) for 1 h prior fixation. BrdU is incorporated in cells of the S-phase during the cell cycle, hence labelling dividing cells. Then, we performed an immunostaining using antibodies directed against BrdU and Ki67, another marker for mitotically active cells [7]. We found that ephrinA5-Fc stimulation leads to increased proliferation, reflected by elevated proportion of cells that incorporated BrdU, as well as by augmented proportions of Ki67 positive cells (Figure 3a–d).

To monitor ephrinA5-induced effects on motility, a function well described in other neuronal subtypes [2,3] as well as cancer cells [29], we analyzed the migratory speed of ephrinA5-Fc and control-treated CB cells in live cell imaging experiments over a time period of 24 h. As depicted in Figure 3e–g, we found increased velocities for migrating CB cells that were treated with ephrinA5-Fc compared to control conditions. In sum, ephrinA5-triggered Eph receptor activation induces physiological effects in immortalized CB cells, which are in line with the triggered gene expression changes of protein-coding genes found in CB cells.

### 2.3. The lncRNA Snhg15 Is Regulated by EphrinA5-Fc and Is Associated with Proliferation of Cancer Cells

**Figure 4:**
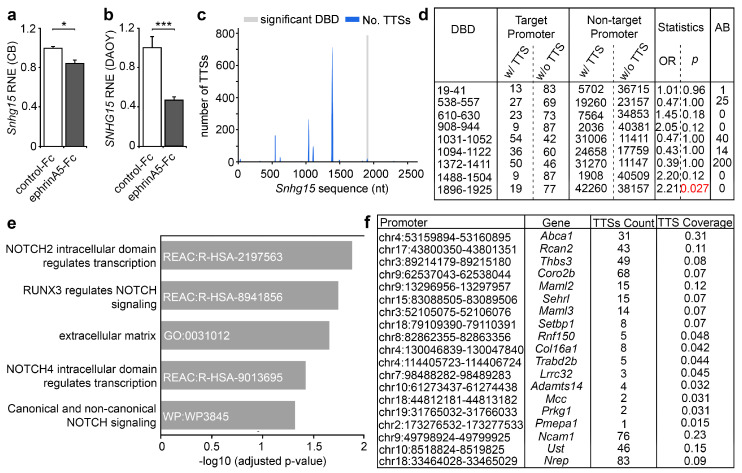
Analysis of *Snhg15* expression and potential triplex target DNA sites (TTS) of *Snhg15* in genes found differentially expressed upon ephrinA5-Fc stimulation. (**a**) Relative normalized expression (RNE) of *Snhg15* in CB cells after ephrinA5-Fc stimulation compared to control-Fc treated CB cells. Expression data and adjusted *p*-value obtained from RNA sequencing of nuclear enriched RNA of CB cells. Sequencing was performed as *n* = 3 biological replicates per condition. (**b**) Quantitative real time PCR analysis of expression of *SNHG15* in DAOY cells treated with control-Fc or ephrinA5-Fc. Data are depicted as relative normalized expression (RNE) of ΔΔCt calculation (*n* = 12 per condition, N = 4 biological replicates). (**c**,**d**) One significant functional DBD (DNA-binding domain) was identified for *Snhg15*, ranging from nucleotides (nt) 1896-1925 (*p* = 0.0274). This site reveals significant predicted binding to the promoter regions of 19 genes that were found significantly up-regulated among ephrinA5-Fc stimulation in CB cells. (**e**) Functional profiling analysis of the 19 putative *Snhg15* target genes (g.Profiler; https://biit.cs.ut.ee/gprofiler/gost) revealed significant enrichment of extracellular matrix and NOTCH signaling-related genes. (**f**) Table collecting the promoter regions and names of genes identified as putative triplex target DNA sites (TTS) of *Snhg15.* Two-tailed Student’s *t*-test, * *p* < 0.05, *** *p* < 0.001.

Among the lncRNAs we identified to be modulated in expression by ephrinA5 stimulation in CB cells, we found *Snhg15*, which was significantly reduced in expression (Figure 2a,b and Figure 4a, Supplementary Table S1). A reduction in expression of human *SNHG15* was likewise observed when stimulating DAOY cells with ephrinA5-Fc (Figure 4b), that similar to CB cells show prominent *EPHA2* expression and ephrinA5-Fc-binding sites (Figure 1c–e). As *SNHG15* affects the expression of genes with roles in cell proliferation, migration, and survival [18], and as these processes have been triggered by ephrinA5-Fc in CB cells, we postulated that *Snhg15* could act as a downstream effector of ephrinA5 signaling. LncRNAs can act at transcriptional and post-transcriptional level. Thus, the modulation of lncRNA expression by ephrinA5-induced signaling represents an effective way of how different genes, that are associated to a particular physiological response, such as proliferation and migration, can be targeted. 

In addition to its function as a competitively endogenous RNA (ceRNA), sponging miRNA in human cancers [19], *Snhg15* was described to be localized and to act in the nucleus, e.g., by interacting with EZH2 that catalyzes repressive trimethylation at histone 3 (H3K27me3) [20]. Hence, we postulated that *Snhg15* modulates transcription at epigenetic level by binding to discrete gene promoters, thereby influencing the expression of respective genes e.g., by interfering with the association of particular chromatin modifiers.

Following this hypothesis, we analyzed potential triplex target DNA sites (TTS) for *Snhg15* in genes found differentially expressed upon ephrinA5-Fc stimulation by using the computational method triple helix domain finder [15]. Indeed, we determined in the *Snhg15* lncRNA a DNA-binding domain (DBD) ranging from 1896 bp–1925 bp, corresponding to chromosomal location chr11:6530448-6530477 (Figure 4c,d, Supplementary File S1). This DBD displays significant binding to promoters of 19 genes found significantly up-regulated upon ephrinA5-Fc stimulation (Figure 4c,d). As *Snhg15* itself was found down-regulated upon ephrinA5-Fc treatment (Figure 4a), we hypothesize that *Snhg15* suppresses the expression of these genes upon promoter binding. As determined by functional profiling, these 19 genes show a significant enrichment of extracellular matrix-associated genes, such as *Adamts14*, *Col16a1*, *Thbs3*, *Lrrc32*, and *Ncam1* (Figure 4e,f). All of these genes have been associated with tumorigenic processes including proliferation and migration regulation [34,35]. While the extracellular matrix is relevant for migration, Notch signaling regulates proliferation of neural stem cells [36] and tumor cells [37,38]. Reactome analysis revealed a significant enrichment of Notch signaling pathways among the *Snhg15* target genes (Figure 4e). Notch signaling is likewise implicated in diverse aspects of cancer biology [37,38]. Thus, ephrinA5 could modulate the transcription of cancer-related genes by acting on *Snhg15* expression as a key lncRNA. 

## 3. Discussion

Sensing and integrating environmental information essentially influence diverse physiological processes in health and disease. Eph receptors and their ligands are expressed in nearly every organ including the brain, fulfilling critical roles during development, such as cell adhesion [2], proliferation [7], neuronal migration [3,39], and axonal guidance [32,40]. Moreover, they are expressed by various cancer cells and in the tumor microenvironment [8], playing a pivotal role in glioma development and progression [1]. Dysregulated expression of ephrins and their receptors is often associated with higher tumor grade and poor prognosis, whereby they can act as both tumorigenic and tumor suppressive. This depends critically on the downstream pathways generated, which in the case of Eph/ephrin signaling is rather complex. Apart from ephrin-elicited forward signaling in the Eph receptor expressing cells, Eph-binding to ephrin-ligands can trigger reverse signaling in the ephrin-expressing cells [8]. Moreover, ligands and receptors can be co-expressed by the same cell, whereby such a ligand-receptor interaction in *cis* modulates the signaling induced in *trans* between ephrins and Ephs expressed by different cells [41]. 

Eph receptor forward signaling triggered by binding of the cognate ligands leads to the activation of numerous pathways that signal to the nucleus, such as the PI3K-Akt/PKB and the Ras/MAPK pathway [9]. These pathways result in gene expression changes that mediate physiological responses. However, the induced alterations in gene expression as well as the gene regulatory mechanisms in the nucleus, which are triggered by Eph receptor activation in the plasma membrane, have received sparse attention so far. Some proof for transcriptional regulation by ephrin-induced signaling arose from microarray-based analysis of postnatal cortical tissue of mice deficient for *efnA5* coding for ephrinA5. The authors observed significant changes in the expression of numerous transcripts with a significant enrichment of genes associated to neuronal circuit development [42]. Further evidence for the gene regulatory potential of ephrinA5 in neurons came from a study of Meier et al. (2011), showing that ephrinA5 modulates gene expression by suppressing the BDNF-evoked neuronal immediate early gene response [43]. Moreover, ephrinA1 affects hepatoma cell growth by regulating the expression of genes related to cell cycle (p21), angiogenesis (angiopoietin 1 and thrombospondin 1), and cell–cell interactions (Rho, integrin, and matrix metalloproteinases) in cultured hepatoma cells [44]. Although Eph/ephrin signaling has been described to be implicated in various cancers and tumorigenesis [8], the effects on gene expression and the underlying mechanisms remain poorly understood. We here revealed differential expression of proliferation, ECM, and neuronal development associated genes upon ephrinA5-Fc stimulation in CB cells, which is supported by increased proliferation and migration as discrete physiological responses. In these cells we identified EphA2 to be most prominently expressed, therefore likely acting as the ephrinA5-interacting receptor. Our findings might be of relevance for medulloblastoma, as one subgroup of medulloblastoma with constitutive activation of the SHH/PTCH pathway has been shown to originate from granule cerebellar progenitors in the external granule layer (EGL), for which immortalized CB cells might be used as a model system for medulloblastoma [24,25]. Prominent EphA2 expression and ephrinA5-Fc binding was likewise observed for DAOY cells, which represents a human tumorigenic cerebellar medulloblastoma cell line (ATCC^®^ HTB-186™). This underlines the relevance of our findings for glioma pathophysiology.

EphA2 is the most frequently affected of all Eph receptors in human cancer. It is often found overexpressed, being often associated with late stage malignant progression and poor prognosis [8,45]. Overexpression of EphA2 was also observed in human glioblastoma multiform (GBM) tumors [28], even accounting as a novel molecular marker and target in GBM [28]. In line with this, down regulation of receptor expression with *EphA2*-specific siRNA oligos retarded tumor growth in pancreatic adenocarcinoma-derived cells [46] and significantly reduces malignancy in glioma [47], non-small cell lung cancer (NSCLC; [48]), and breast cancer cells [49]. For the more demanding in vivo application the nanoliposomal EphA2-targeted therapeutic EPHARNA (1,2-dioleoyl-sn-glycero-3-phosphatidylcholine; DOPC) was developed, leading to efficient reduction in EphA2 expression and tumor growth in an ovarian cancer mouse xenograft model [50].

In this study we provided evidence that ephrinA5-Fc stimulation induces increased expression of EphA2 in DAOY cells, suggesting that Eph receptor activation by cognate ligands, e.g., presented by the microenvironment, could trigger the overexpression observed in glioma [28]. In stem-like tumor-propagating cells from human glioblastoma, the EphA2 receptor drives the self-renewal and tumorigenicity [51]. EphA2 further promotes infiltrative invasion of glioma stem cells [52]. However, in addition to pro-oncogenic functions of EphA2, tumor suppressor functions were likewise described [8,45]. 

Similarly, ephrinA5 is known to regulate focal adhesion, cell motility, and cancer invasion by remodeling of the actin cytoskeleton [53,54]. Similar functions were described for the developing nervous system [4]. An ephrinA5-dependent modulation of proliferation and migration was already reported for cells with tumor initiating properties isolated from a mouse model of gliomagenesis, that relies on the loss of PTEN and p53, which represent two common genetic alterations in wild-type GBM [55]. In contrast to these cells, we found an increase in proliferation and motility in our cell culture model. This finding is supported by gene expression analysis that revealed a significant reduction of cell death-associated genes upon ephrinA5-Fc stimulation.

Apart from protein-coding genes, we revealed numerous ncRNAs to be modulated in expression by ephrinA5-Fc-induced signaling. Among these, we found *Snhg15/SNHG15* was significantly downregulated in both CB and DAOY cells. In humans, the lncRNA *SNHG15* has been associated with diverse cancer types, such as breast cancer, colorectal cancer, hepatocellular cancer, lung cancer, osteosarcoma, ovarian cancer, thyroid cancer, pancreatic cancer (PC), renal cell carcinoma, in addition to glioma [13]. Thereby, *SNHG15* was reported to have both cancer- and metastasis-promoting and also tumor-suppressing functions, suggested to be linked with poor survival in numerous human malignancies [13]. In breast cancer, elevated levels of *SNHG15* were associated with proliferation, migration, and invasion of breast cancer cells through acting as a competing endogenous RNA (ceRNA) to sponge miR-211-3p [13]. Similarly, in glioma vascular endothelial cells *SNHG15* expression levels were notably upregulated compared with primary astrocytes [56]. 

In contrast, Liu et al. found *SNHG15* downregulated in 50 thyroid cancer tissues and four cell lines [57]. Detailed correlations with several parameters such as age, pathology classification, clinical stage, tumor size, and others, lead the authors to conclude that *SNHG15* rather acts as an anti-tumor gene in thyroid cancer. They further found that elevated *SNHG15* suppressed proliferation, migration, and invasion significantly. Inhibition of *SNHG15* by miR-510-5p was revealed to promote cell proliferation, migration, and invasion in thyroid cancer [58].

Similarly, we determined a negative correlation between *Snhg15* expression and proliferation/migration of murine CB cells, suggesting that the function of *Snhg15* critically depends on the cell types investigated. Further, our data propose a novel mechanism of *Snhg15* function, acting in the nucleus capable of forming triplex structures with gene promoters found contrarily regulated to the *Snhg15* expression. LncRNAs have frequently been reported to act on transcription by evicting or promoting the binding of chromatin modifiers to discrete gene loci, being able to act in *cis* and *trans* [14]. Hence, transcriptional control by *Snhg15* via triplex structure formation with distinct promoters might be an additional mechanism through which *Snhg15* could be involved in transcriptional regulation of underlying cancer-related processes. 

Among the genes being up-regulated upon ephrinA5-Fc stimulation and displaying putative binding sites for *Snhg15* in their promoter region, we identified a significant enrichment of extracellular matrix-related genes such as *Ncam1* and *Adamts14*. NCAM1 is a member of the immunoglobulin superfamily that mediates both homophilic (NCAM to NCAM) and heterophilic binding (NCAM to sulfate proteoglycans or other collagens), hence being crucial for cellular interactions and migration [59]. Overexpression of NCAM and its polysialylated form (PSA-NCAM) have been observed in diverse metastatic cancers, including neuroblastoma [60]. Increased NCAM expression levels were shown to directly cause a loss of adherens junctions and the initiation of tumor invasion [61]. Moreover, ADAMTS14 belongs to the family of metalloproteases (ADAMTSs), which represent complex extracellular proteases that have been associated to both oncogenic and tumor-protective contexts [34]. The other ECM-related genes (*Col16a1*, *Thbs3*, *Lrrc32*) are likewise implicated in tumorigenic processes such as proliferation and migration [62,63,64]. Proliferation is further regulated by NOTCH signaling in both neural stem cells [36] as well as tumor cells [38]. Interestingly, among the genes found up-regulated in response to ephrinA5-Fc stimulation, we further identified two important activators of the NOTCH-signaling pathway, namely mastermind-like 2 (*Maml2*) and mastermind-like 3 (*Maml3*) as putative *Snhg15* targets. Both are known to promote proliferation of different cancer cells [65,66,67]. 

Although more detailed investigations in discrete glioma cell types are required, we here provide novel findings, showing the ephrinA5-induced signaling activates gene expression programs that promote cell proliferation and migration. This could be mediated by the ephrinA5-dependent transcriptional repression of an important cancer-related lncRNA, namely *Snhg15*, which might lead to the increased expression of cancer-relevant protein-coding genes, putatively by the loss of *Snhg15* triplex binding in gene promoters. 

## 4. Materials and Methods 

### 4.1. Cell Culture and Treatment with Recombinant EphrinA5-Fc

CB cells [68] were cultured in Dulbecco’s modified Eagle’s medium with high glucose (DMEM, #41965-039, Thermo Fisher Scientific, Waltham, MA, USA) supplemented with 10% FBS (Biowest, Nuaillé; France), 1X GlutaMAX, 28 mM KCl at 33 °C, 5% CO_2_, and 95% relative humidity. Upon thawing of cells, the medium additionally contained 100 U/mL penicillin and 100 µg/mL streptomycin until the first passage. The cells were split after reaching a confluence of 75%. DAOY cells (ATCC^®^ HTB-186™) were cultured in minimum essential Eagle’s medium with EBSS (MEM, # P04-08058, Pan Biotech, Aidenbach, Germany) supplemented with 10% FBS (Biowest, Nuaillé; France), 2 mM L-Glutamine, 1 mM sodium-pyruvate, 1.5 g/L NaHCO_3_, 100 U/mL penicillin, and 100 µg/mL streptomycin incubated at 37 °C, 5% CO_2_, 17% O_2_, and 95% relative humidity. For stimulation, both cell lines were treated with 5 μg/mL ephrinA5-Fc (R&D Systems, Minneapolis, MN, USA) or Fc protein as control (Rockland Immunochemicals, Gilbertsville, PA, USA) pre-clustered with 10 μg/mL Alexa488 conjugated anti human IgG (Thermo Fisher Scientific, Waltham, MA, USA) for 30 min at RT and then applied to the cells. For proliferation assays, 3 µg/mL BrdU was applied 5 h after ephrinA5-Fc or Fc stimulation and one hour prior to fixation with 4% paraformaldehyde in phosphate buffered saline (PBS). For migration assay cells were treated with ephrinA5-Fc or Fc and live cell imaging was performed for 24 h.

### 4.2. Immunocytochemistry 

Fixed CB and DAOY cells were washed three times with 1× PBS/0.5% Triton X-100 for 10 min for BrdU and Ki67 staining and with 1× PBS/0.2% Triton X-100 for 5 min for ß3-tubulin staining prior to blocking with 1× PBS/0.2% Triton X-100/4% BSA for 30 min or 5% normal goat serum in 1× PBS/0.5% Triton X-100 for 1 h, respectively. For BrdU and Ki67 staining an antigen retrieval with citrate buffer at 90 °C for 15 min and a HCl treatment (1 N HCl at 4 °C for 30 min followed by 2 N HCl for 30 min at room temperature and subsequent incubation in 10 mM borate buffer, pH 8.0 for 10 min) was conducted. After washing, primary antibodies were applied overnight at 4 °C. After washing four times with respective washing buffer for 10 min, secondary antibody was applied for 3 h at room temperature prior to DAPI staining (Thermo Fisher Scientific, Waltham, MA, USA) for 5 min. The following primary antibodies were used: rabbit anti Ki67 (Linaris, Germany; 1:500); rat anti BrdU (Bio-Rad Laboratories, München, Germany; 1:500); rabbit anti ß3-tubulin (Merck KGaA, Darmstadt, Germany; 1:2000). After washing (three times), the following secondary antibodies were applied: goat anti-rat Cy5 (Thermo Fisher Scientific, Waltham, MA, USA; 1:1000), goat anti-rbt Cy3 (Jackson Immunoresearch, West Grove, PA, USA; 1:750).

### 4.3. Migration Assay

Standard TC cell culture plates (Eppendorf, Hamburg, Germany; 170 µm glass thickness) were coated with matrigel (GelTrex™; Thermo Fisher Scientific, Waltham, MA, USA) according to the manufacturer’s instructions with a final concentration of 0.2 µg/mL GelTrex^TM^ diluted in 1 mL of CB culture medium without phenol red. The GelTrex^TM^ working solution was applied to each well and was incubated for 60 min at 33 °C. Cells were seeded with a density of 54 cells/mm^2^ and incubated for 24 h at 33 °C. To avoid phototoxic effects during imaging, culture medium was exchanged to phenol red free DMEM (Thermo Fisher Scientific, Waltham, MA, USA) with all ingredients. Then, cells were treated with 5 µg/mL efnA5-Fc or control-Fc, pre-clustered with 10 µg/mL anti human IgG antibody. Imaging was started immediately afterwards. CB cells were imaged every 20 min for 24 h at 33 °C and 5% CO_2_. 

### 4.4. Microscopy and Data Analysis

Live cell imaging was conducted using a DMi8 inverted microscope equipped with the thunder imaging platform (Leica, Wetzlar, Germany). For analysis of migration distances, image processing was done with the Basic default plugin from Fiji (vers.2.0.0-rc-69; [69]). Binarization of corrected brightfield images was performed using a Pyton-based edge detection algorithm. These binary image sequences were analyzed with the 3D-analysis suite for migratory behavior of the cells (Leica, Wetzlar, Germany, LasX software module). Representative cells were converted to videos, color code, and panel illustration using Fiji ImageJ [69]. For evaluation of the migration range, all cells were included, which had a migration time of at least 6 h. As a demonstration of the different migration ranges, five different timepoints (0 h, 3 h, 6 h, 9 h, and 12 h) of a respective cell were selected and a temporal color code over the same 12 h period was added.

For immunocytochemistry analysis images were likewise acquired by the DMi8 inverted microscope equipped with instant computational clearing for confocal like imaging (Leica, Wetzlar, Germany). Photographs were analyzed using the free Fiji software [69]. For fluorescence intensity-based thresholding for the automated analysis of Ki67 and BrdU labeling, each experimental design was imaged with the same settings regarding exposure time and light intensity. Leica LasX 3D analysis module was used to determine the thresholds and measure the fluorescent signal of Ki67 and BrdU within the nucleus using DAPI signal as the reference mask. Positive signals were filtered by integrated density and presented as percentage of positive cells in regard to DAPI positive cells. Photoshop CC (Adobe Inc., San José, CA, USA) was applied for image illustration. Boxplots were plotted using R and barplots using excel (Microsoft, Redmond, WA, USA). Significance was analyzed with two-tailed Student’s *t*-test or two-way ANOVA. Significance levels: *p* value < 0.05 *; *p* value < 0.01 **; and *p* value < 0.001 ***.

### 4.5. Nuclear Enrichment and RNA Isolation of CB Cells

Medium was removed and cells were washed with cold 1x PBS (pH 7.4). Total of 500 µL of cold nuclei buffer (10 mM Tris-HCl pH 7.9; 100 mM KCl 5 mM MgCl_2_) was added after removing PBS and incubated for 5 min on ice. About 20 µL of 10% NP40 and 0.5 µL DTT were added and were shaken at 200 rpm for 2 min on ice to perform cell lysis. Suspension was transferred to a 1.5-mL tube and centrifuged for 2 min at 1200× *g* at 4 °C. After discarding of supernatant, 500 µL of nuclei buffer was added for pellet resuspension. After centrifugation for 2 min at 200× *g* at 4 °C, supernatant was discarded. Pellet was washed twice with 125 µL of glycerol buffer (20 mM Tris-HCl pH 7.4; 50% glycerol; 75 mM KCl; 1 mM DTT; 5 mM MgCl_2_) with centrifugation for 5 min at 500× *g* at 4 °C. Finally, supernatant was discarded and pellet resuspended in 50 µL of nuclei buffer. After adding 450 µL of Trizol^®^Reagent (Thermo Fisher Scientific, Waltham, MA, USA) all samples were snap frozen on dry ice and stored at −80 °C. For RNA-sequencing the samples were subjected to standard RNA isolation procedure using Trizol^®^Reagent with additional application of GlycoBlue (Thermo Fisher Scientific, Waltham, MA, USA) to a final concentration of 0.2% during RNA precipitation for better visualization of the pellet. RNA and DNA quantities were determined using Qubit 4 Fluorometer (Thermo Fisher Scientific, Waltham, MA, USA). 

### 4.6. cDNA Synthesis and Quantitative PCR

RNA was isolated with Trizol^®^Reagent (Thermo Fisher Scientific, Waltham, MA, USA) according to manufacturer’s guidelines and was used for cDNA synthesis using iScript cDNA synthesis kit (Bio-Rad Laboratories, München, Germany). Quantitative PCR reactions were performed using 10 ng cDNA of each sample and the PowerUP SYBR Green qPCR Kit (Thermo Fisher Scientific, Waltham, MA, USA) on the CFX96 thermocycler (Bio-Rad Laboratories, München, Germany). The primer sequences are listed in Supplementary Table S2. Data analysis was performed using ΔΔCt calculation with reference genes *Rps29* (for mouse CB cells) and *RPS18* (for human DAOY cells). Relative normalized expression was calculated in regard to control Fc-treated samples.

### 4.7. RNA Sequencing 

Library preparation for RNA-Seq was performed using the NEBNext^®^ Ultra™ II Directional RNA Library Prep Kit for Illumina^®^ (New England Biolabs, Ipswich, MA, USA, E7760) starting from 36 ng of total RNA as input. Ribosomal depletion was achieved by using the NEBNext^®^ rRNA Depletion Kit (Human/Mouse/Rat) (New England Biolabs, Ipswich, MA, USA, E6310) and indexing was performed by the use of NEBNext^®^ Multiplex Oligos for Illumina^®^ (New England Biolabs, Ipswich, MA, USA, E7335). Accurate quantification of the input RNA was performed with the QuantiFluor^®^ RNA System (Promega, Madison, WI, USA, E3310) and by TapeStation RNA ScreenTape Analysis (Agilent, Santa Clara, CA, USA, 5067-5576). Accurate quantification of cDNA libraries was performed by using the QuantiFluor™ dsDNA System (Promega, Madison, WI, USA). The size range of final cDNA libraries was determined applying the TapeStation D1000 ScreenTape (Agilent, Santa Clara, CA, USA, 5067-5582). cDNA libraries were amplified and sequenced using the NextSeq 500 with NextSeq 500/550 High Output Kit v2.5 (150 cycles; Illumina, San Diego, CA, USA, 20024907). Sequencing was performed as paired end; 2 × 76 bp; single indexing with ~30 million reads per sample. 

### 4.8. Alignment, Quality Control, Batch Effect Correction, and Differential Expression Analysis

The paired-end FASTA files have been processed by the nextflow pipeline nfcore/rnaseq v1.4.2 mainly using the standard settings [70]. The only changes that have been made to the default settings are specifying the strandedness of the library as reverse stranded and setting the aligner and pseudoaligner tools. The nfcore/rnaseq pipeline used Cutadept v2.9 for adapter trimming and executed an extensive quality control that was summarized in a MultiQC report by MultiQC v1.7. The reads were aligned to the reference genome MM10 using STAR v2.6.1d. The gene counts were determined by using featureCounts v1.6.4. Gencode M24 was used as a reference transcriptome for both alignment and counting. In addition to the alignment, a pseudoalignment providing transcript expression quantification was performed with Salmon v0.14.1. Next, we performed a Voom transformation of the RNA-seq count matrix adjusting for sample importance. The differential expression was analyzed with limma v3.40.6. This includes the application of TMM normalization and the removal of the observed batch effect by including the batch variable in the design of the fitted linear model. Genes with positive logFC are up-regulated and those with negative logFC are down-regulated in ephrinA5-Fc treated samples. Genes having a Benjamini-Hochberg adjusted *p*-value < 0.05 were considered as significantly differentially expressed.

### 4.9. LncRNA Binding Prediction

For *Snhg15* possible triplex-forming sites and binding domains inside of promoters of other differentially expressed genes were identified using the TDF promoter test from the RGT toolbox v0.13.1 [15]. As input for this tool a single sequence FASTA file with *Snhg15* sequence was provided containing the sequence of all exons of all transcript isoforms. This was created using bedtools v2.26.0 (Supplementary File S1). Exon data for individual transcripts were retrieved from UCSC Genome Table Browser and the exons were combined with bedtools merge in a strand-specific manner. The exon sequence was extracted from the reference genome Gencode M24 GRCm38.p6 with bedtools getfasta. The TDF promoter test was applied independently to the differentially up- and down-regulated genes with organism specified as “mm10” and the minimal length of reported binding sites was set to 15 bp.

## Data Availability

Sequence data will be deposited in NCBI’s Gene Expression Omnibus and are accessible through GEO Series upon acceptance of the manuscript.

## Supplementary Materials

The following are available online at https://www.mdpi.com/1422-0067/22/3/1332/s1. Supplementary Legends; Supplementary Figure S1—RNA sequencing of nuclear enriched and ribosomally depleted RNA of CB; Supplementary File S1—Snhg15 master transcript; Supplementary Table S1—sign DEG efnA5 vs ctrl; Supplementary Table S2—Primer.
