# Supplementary material for: The Expression of the Cancer-Associated lncRNA Snhg15 Is Modulated by EphrinA5-Induced Signaling
Source: Int J Mol Sci. 2021 Jan 29;22(3):1332. doi: 10.3390/ijms22031332 (PMC7866228; doi:10.3390/ijms22031332)
Supplement: Supplementary file 1 [file ijms-22-01332-s001.zip › Supplementary Informations/Supplementary File S1 - Snhg15 master transcript.docx]

**Legend:**

Exon – Exon boundry

**Non-significant DBD in the promoters of sign. up-regulated genes**

**significant DBD in the promoters of sign. up-regulated genes**

ATGCAGAAATGGAGACTC**CAGAGCTGGAGAGAGAGAGATGAC**TCAACTGTTAAAAGCACTGACTGCTCTTCCAGAGGTCCTGAGTTCAATTCCCAGCAACCACATGGTGGCTCACAATTATCTGTAATGGGGATCCGATGCCCTCCTCTGGTGTGTCTGAAGACAGTGATGGTGTACTGACATATATGAAGTAAATAAGTAAATTAATAAATAGAAATGAGACTCCAGGCTCGCTGCTGTGACCGTAACTTTAAGCACACGTGAGCGTCCTCAGCTGGCAGCCATGGCTGGCGGGTCCTTCCTAGCATGTGCTGCGGCCCTCACCTTATCTTCCGTCATCAGGGCACAGGCGCCGTGCAGGGCTATTCCGAGTCCAGGTAGAGATGGCTGAGCTCGGTGGCAGGAGATCAGCCTGCGGTGGAGCAGGGACGTTTGCCCTGGGGAGACACGTGCCACCGGGTGAGTTAGGCCTGTTTCTGGGGGATTCCTTTCCAACCCCAGCTAGTTCCGCCCAAGCAAGCAGAGTCTTCCCTCAAG**AGATTTTTTTTTCCTTTTAC**GAGAGAACGTTCGGAGGGGCTAGAACTGGGTGATACGCACTGGCTGCGCTGA**ATCTGTCTTTTCTCCCCTCCT**AGGTGGACATCGAGGCCCTGTGGGTGGCCCTACAGCGTGGAAGGGATGACCGAGCTTTGGGACATGTCCTGGTAAGTCAGTTGTGGAAGCTCGGTGGCCGGTGAAGCCAAACTCCGTATCTTGAATTCATTTTCAGGACTAATTCTCTCCCCTAGACTTCTGGGGACTGGGAGACTGCAGTATGGGTCGTGCAGGATTGGAGTGATATACTTAGCAAGCCTCCAGCGTGCTTGGGTCTGCAGTGACCCTG

TGCATTCCTACAGTGCTTGCCAGAACA**ATTTTGAAGTGGTTTGAGGCCTTGCCCTGCCCTCTCC**AGAGCAAGGTTATAGAAATTTCAGACAATATGGCAGACACCTGCCACGTGGATAAATTACAAGCCGGTAAGATTTGCAATGCTGCA**CTTTGGGTTTTTTGTTTTGTTT**AACTGTGGGGATAGTTCTGCACATGGTGCAGAGGCAAATA**AGTCATTTCTTGTTGGTTTTGTTTTGAGG**CAAGGTTTCTCTGTAGTTCTTGCTGTCCTGGAACTCAAAACAGATCCACTCACCTCTGCCTCCTGAGTGCTGGGATTAAAGGTGTGTAAGGCTACACCCAGCTGAATATTTTTCTGTGCTCCAGAGTTAGTACAAAGTAGTAAATCCTTAAAACAGTTACAAAATACAGCAGGAGTACCAGAGTTGGGTCTCCGGTGTTGAAGTAAAACCCCAGGTGTGGTGACACGTGGTTTTGGTTCCATTTGGGCTC**TAGGCCTTATTTTTCTTTTTTTTTTTTTCTTTTCTCGAG**ACAAGGTTTCTCTGTGTAGCCCTGGCTGTCCTGGAACTCACTTTGTAGACCAGGCTGGCCTGGAACTCAGAAATCC**ATCTGCCTCTACCTCCC**GAGTGCTGGGATTAAAGACGTGTGCCACCACACCGGGCCTTAGGCCTGGTTCTTAAAAGGCAGAGAGCCTGCAGATGGTAGAAATCTGGCTGCTGTGCCAATACCTAAGGAAAGCCTCCCGGCAAAAGGAAGGAATCCCAGGTACTGGGATTAAGGGTGTGTGTTACCACACCAGGCCCTCATTTCAAATCTTTATGTTGAAAGGGAATAAAACTGTAATTCATGTAATGTATGTTAAATAGCCCAAAGAGTTGTTTCTGAGCTTTGAAACCTGGGGCTGAGAACATAGCAGAACAGACCAGGACATGCCCGGGCAAGCCCATCGCCTCCCTAGCTCCCACCCCTCTGACCTAAGTTAAATGTTACAGGCTGCTGATGTGAAACCGCGCCA**ATTCCTCCCCCAGCCCCACTCCTTTTCTAT**AAAAACCCCTAGCTTCCAAGCCTCGTGGTCGAATCCACTGTCTCCTGTTATGTGAGATACGTTTCGACCCGGAGCTCCGCCATTAAAAAAACCTCTTGTTGTTACATCAAGGTGTTGTGTTCTATTCGTGATTCTTGGGTGCACGCCGAATCGGGAGCTGAGTGGGGGTTTCCCCACTGAGTTCTTTCAATGTCACATATGTGAGTGCACTGTCACTGACTTCAGACACACCAGAAGAGGGCATCAGATCCCATTACAGATGGTTCTGAGCCACCACGTGGTTGCTGAGTTCAGGACTTCTAGAAGAGCAGTCAGTGATAACTGCTGAGCTATCTATCTCCAGCCCCTTAACTCAAACCTTTAATTGTACTTCTTTTGTGTGCGTGTCCATGGGTAGGTATGGGCCAATGGATGGCATAGAACATCAAGGTCAGAGAACAGCTTGCAGGAATTGGTTTTCTCCTTTGATAGCAGGCATCTCTAGTCCTACAGCCTGTGTTCTTTTCTGGAGTGCCAGTCATGACTGAGTTGTGTTGAAGGGAAATGTCCTGTTCTTGGTATGGAAGCTGTGGGGTAAATGGTACGTGGACAGTTGCCAGCTTAGTGTTAATGCATTTGGCTCACTGACTCTTCCTTCGGTCTAGGAAACCTAAGGTGCTGGAGCTAGAAGAGACTTGATAGCACTTCAGAGACCATCAGGCTGTTTCGTGGAACTTCCACCAAAGAG
