# Supplementary material for: The Expression of the Cancer-Associated lncRNA Snhg15 Is Modulated by EphrinA5-Induced Signaling
Source: Int J Mol Sci. 2021 Jan 29;22(3):1332. doi: 10.3390/ijms22031332 (PMC7866228; doi:10.3390/ijms22031332)
Supplement: Supplementary file 1 [file ijms-22-01332-s001.zip › Supplementary Informations/Supplementary Legends.docx]

**Supplementary Figures:**

**Supplementary Figure S1 - RNA sequencing of nuclear enriched and ribosomally depleted RNA of CB cells.tif**

RNA sequencing of nuclear enriched and ribosomally depleted RNA of CB cells, treated either with ephrinA5-Fc or control Fc. **(a)** Principle Component Analysis (PCA) plot after batch correction, with control Fc samples shown in black and ephrinA5-Fc treated samples shown in red. Principle Component Analysis (PCA) has been done with FactoMineR v2.3. The PCA results have been visualized by plotting PC2 against PC1. **(b)** Pie chart illustrating the proportions of read annotations for protein coding genes, lncRNAs, other non-coding RNAs and others, showing high read numbers for lncRNAs. **(c)** Proportional distribution of annotation for protein coding genes, lncRNAs, miRNAs, pseudogenes and other biotypes of all detected genes. **(d)** Heatmap illustrating the log2 fold change of the differentially expressed protein coding genes upon ephrinA5-Fc stimulation in CB cells.

**Supplementary Material:**

**Supplementary File S1 - Snhg15 master transcript.docx**

Master transcript for Snhg15 of all isoforms retrieved from UCSC Genome Table Browser. Exons were combined with bedtools merge in a strand-specific manner. All predicted but non-significantly enriched DNA-binding domains (DBD) within the sign. up-regulated genes are highlighted in grey. The significantly enriched DBD with binding regions within the promoter regions of sign. up regulated genes within the ephrinA5-Fc treated CB cells is highlighted in red.

**Supplementary Table S1 – sign DEG efnA5 vs ctrl.xlsx**

List of all significant differentially expressed genes between control-Fc and ephrinA5-Fc treated CB cells. Genes with positive logFC are up-regulated and those with negative logFC are down-regulated in ephrinA5-Fc treated samples.

**Supplementary Table S2 – Primer.xlsx**

List of qRT primers including product size used for DAOY (human) and CB (mouse) cells.
