# Supplementary figures and images for: The Expression of the Cancer-Associated lncRNA Snhg15 Is Modulated by EphrinA5-Induced Signaling
Source: Int J Mol Sci. 2021 Jan 29;22(3):1332. doi: 10.3390/ijms22031332 (PMC7866228; doi:10.3390/ijms22031332)

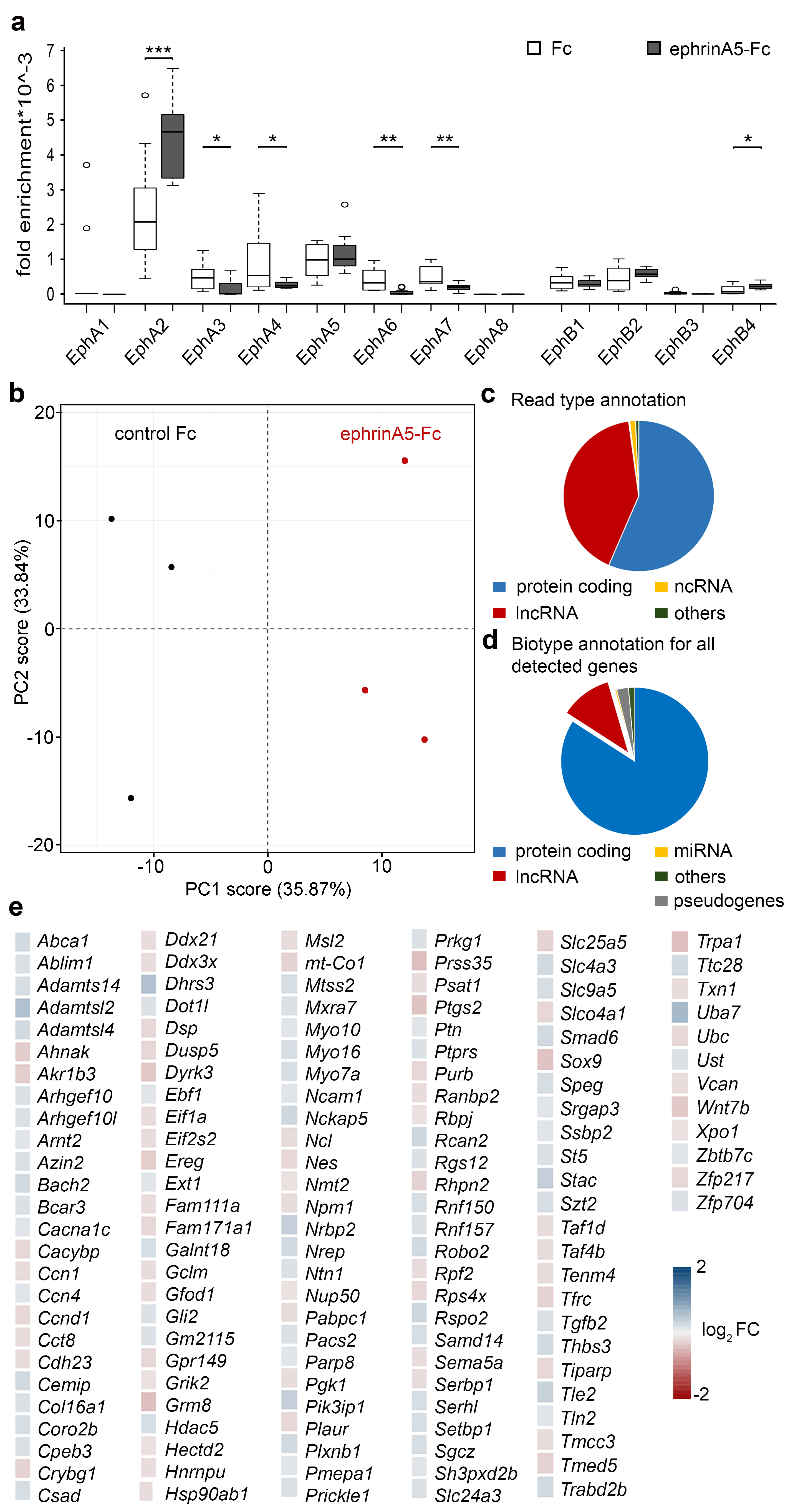

Supplement: Supplementary file 1 [file ijms-22-01332-s001.zip › Supplementary Informations/Supplementary Figure S1 - RNA sequencing of nuclear enriched and ribosomally depleted RNA of CB.tif]
